# Process development for scale-up production of a therapeutic L-asparaginase by *Streptomyces brollosae* NEAE-115 from shake flasks to bioreactor

**DOI:** 10.1038/s41598-019-49709-6

**Published:** 2019-09-19

**Authors:** Noura El-Ahmady El-Naggar, Hassan Moawad, Nancy M. El-Shweihy, Sara M. El-Ewasy, Islam A. Elsehemy, Nayera A. M. Abdelwahed

**Affiliations:** 1 Department of Bioprocess Development, Genetic Engineering and Biotechnology Research Institute, City for Scientific Research and Technological Applications, Alexandria, Egypt; 2 Department of Agricultural Microbiology at National Research Center, Cairo, Egypt; 3 Chemistry of Natural and Microbial Products Dept., Pharmaceutical Industry Division, National Research Centre, 33 El Buhouth St.(Former El Tahrir St.), 12622-Dokki Giza, Egypt

**Keywords:** Applied microbiology, Industrial microbiology

## Abstract

L-asparaginase is a promising enzyme that has a wide range of significant applications including cancer therapy and starchy food industries. The statistical design of Plackett–Burman and face centered central composite design were employed to optimize L-asparaginase production by *Streptomyces brollosae* NEAE-115. As a result, a medium of the following formula is the optimum for producing L-asparaginase in the culture filtrate of *Streptomyces brollosae* NEAE-115: Dextrose 2 g, starch 20 g, L-asparagine 10 g, KNO_3_ 1 g, K_2_HPO_4_ 1 g, MgSO_4_.7H_2_O 0.5 g, NaCl 0.1 g, pH 7, fermentation period 7 days, temperature 30 °C, inoculum size 4%, v/v, agitation speed 150 rpm and inoculum age 48 h. The kinetics of cell growth, carbohydrates consumption and L- asparaginase production were studied in 7-L stirred tank bioreactor under different cultivation conditions. A significant increase in both cell growth and carbohydrate consumption was observed as the stirring speed increased from 200 to 600 rpm under uncontrolled pH. The highest L- asparaginase activity of 108.46 U/mL was obtained after 96 h at 400 rpm. On the other hand, the specific enzyme production (Y_p/x_) under uncontrolled pH reached its maximal value of about 20.3 U/mg cells. Further improvement of enzyme production was attained by controlling pH at 7 using the selected stirring speed of 400 rpm. Enzyme production of 162.11 U/mL obtained from the controlled pH cultures exceeded this value gained from uncontrolled pH (108.46 U/mL) by about 50%.

## Introduction

L-asparaginase (L-asparagine amino hydrolase, EC3.5.1.1) is an enzyme of high therapeutic value due to its use in certain types of cancer therapy mainly in acute lymphoblastic leukemia (ALL)^1^. It is a tetrameric enzyme that catalyses the hydrolysis of L-asparagine to L-aspartic acid and ammonia^2^. The anti-neoplastic activity of L-asparaginase depends on the fact that neoplastic cells require a large amount of L-asparagine as amino acid to keep up with their rapid malignant growth. The neoplastic cells are deficient in L-asparagine synthetase and are unable to synthesize the needed asparagine-dependent proteins. Therefore, they are dependent on the external sources “consumed in the diet, absorbed in the body and available in the serum” to obtain L-asparagine^3^ for their survival and propagation. Conversely, normal healthy cells are able to synthesize L-asparagine and are therefore protected from L-asparagine-starvation^4^. Therefore, the injection of L-asparaginase intravenously, drastically reduces the level of free L-asparagine in the blood stream and leading to the neoplastic cells are selectively killed due to the absence of L-asparagine^5^. Moreover, L-asparaginase is also used in several industrial fields such as food processing to convert L-asparagine to aspartic acid. Before frying or baking, pretreatment of starchy foods with L-asparaginase reduces acrylamide formation (a carcinogenic toxicant)^6^.

Microbial L-asparaginases have been particularly studied for their applications as a chemotherapeutic agent in the treatment of human cancer^7^. L-asparaginase is produced from a variety of microbial sources including fungi, yeast, bacteria and actinomycetes by the process of submerged fermentation^8^. Currently, L-asparaginases derived from two bacterial sources, *Erwinia chrysanthemi* and *E. coli*, are in clinical use for the treatment of ALL^9^. Few actinomycetes were reported to produce extracellular L-asparaginase, while cell bound L-asparaginase was reported in few actinomycetes like *Streptomyces karnatakensis* and *Streptomyces albidoflavus*^10^. Stirred tank bioreactors have been applied for the production of enzymes in large scale batch fermentation^11^.

The aim of the present work targeted the optimization of different process parameters for L-asparaginase production by *Streptomyces brollosae* NEAE-115 using submerged fermentation in the shake flasks and to optimize the cultivation parameters for large scale production in 7-L stirred tank bioreactor.

## Results and Discussion

**Figure 1:**
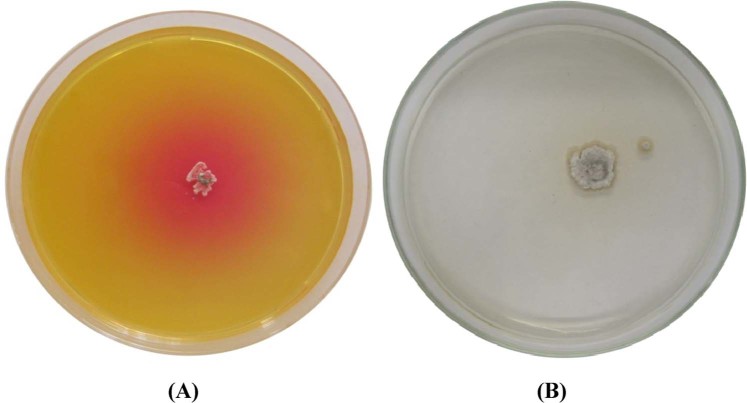
L-asparaginase activity of *Streptomyces brollosae* NEAE-115 detected by plate assay (**A**) production of the enzyme after two days; (**B**) control plate was prepared without dye.

Extracellular L-asparaginase production by *Streptomyces brollosae* NEAE-115 was detected by plate assay method. The color change in the medium from yellow to pink zone surrounding the colony indicated that the organism is a potent producer of L-asparaginase (Fig. 1). In our previous study^12^; L-asparaginase production was performed using submerged fermentation. The enzyme was fractionated by ammonium sulphate precipitation and the purified enzyme was obtained using the ion exchange column of DEAE-Sepharose CL-6B. The purity and molecular weight of the purified enzyme was determined by SDS-PAGE separation, which revealed only one distinctive band with a molecular weight of 67 kDa. The purified L-asparaginase was widely active in pH ranging from 4.5 to 10.5 with maximum activity at pH 8.5. Also, it was widely active at a temperature of 25 to 60 °C with maximum activity at 37 °C at incubation time of 50 min and optimal substrate concentration of 7 mM. The enzyme is free of glutaminase activity. Maximum stability of purified L-asparaginase was observed at 40 °C. The enzyme was more stable in alkaline pH (pH 8.5) than acidic. In addition, mice treated with *Streptomyces brollosae* NEAE-115 L-asparaginase inhibited Ehrlich Ascites Carcinoma cells growth in female Swiss albino mice by 79% with higher cytotoxicity than commercial L-asparaginase. Therefore, in this study, optimization of the various process variables affecting production of L-asparaginase by *Streptomyces brollosae* NEAE-115 was performed using submerged fermentation in shake flasks and 7-L stirred tank bioreactor.

### Evaluation of variables affecting L-asparaginase production by *Streptomyces brollosae* NEAE-115 using Plackett–Burman design

**Figure 2:**
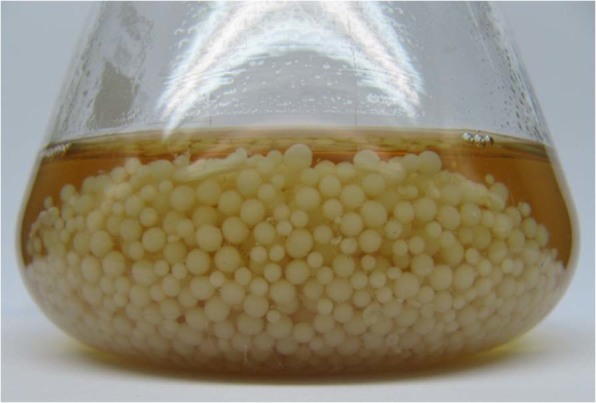
*Streptomyces brollosae* NEAE-115 growth in large spherical pellets during L-asparaginase production in shake flasks.

In order to evaluate the effects of 15 different independent variables and to determine the most significant ones for the maximum production of L-asparaginase, the Plackett-Burman design was applied (Table 1). Dummy variables (D_1_, D_2_, D_3_ and D_4_) were used to calculate the standard errors of the experiment in data analysis. Table 2 shows the Plackett-Burman design matrix selected to screen the significant variables for L-asparaginase production along with the corresponding experimental and predicted L-asparaginase production. The mycelial growth has developed as large spherical pellets during production of L-asparaginase in shake flasks that can improve the L-asparaginase production than free filament growth (Fig. 2). The variation in L-asparaginase activity shown in various Plackett-Burman design runs was markedly wide (6.604 to 79.935 U/mL) and reflected the significance of the optimization process to maximize L-asparaginase production. The maximum L-asparaginase activity (79.935 U/mL) was achieved in the run number 8 when highest levels of incubation time, inoculum size, agitation speed, L-asparagine and lowest levels of inoculum age, initial pH, K_2_HPO_4_, NaCl were used. While the minimum L-asparaginase activity (6.604 U/mL) was obtained in the run number 10 when minimal levels of incubation time, inoculum size, agitation speed, L-asparagine and maximal levels of inoculum age, initial pH, K_2_HPO_4_, NaCl were used.

**Table 1:** Experimental independent variables at two levels used for the production of L-asparaginase by *Streptomyces brollosae* NEAE-115 using Plackett–Burman design.

Code	Variables	Levels	
		−1	+1
X_1_	Temperature (°C)	30	37
X_2_	Incubation time (days)	5	7
X_3_	Inoculum size (%, v/v)	2	4
X_4_	Inoculum age (h)	24	48
X_5_	pH	7	9
X_6_	Agitation speed (rpm/min)	100	150
X_7_	Dextrose (g/L)	2	4
X_8_	Starch (g/L)	10	15
X_9_	L-asparagine (g/L)	4	7
X_10_	KNO_3_ (g/L)	1	3
X_11_	Yeast extract (g/L)	0	1
X_12_	K_2_HPO_4_ (g/L)	1	2
X_13_	MgSO_4_.7H_2_O (g/L)	0.1	0.5
X_14_	NaCl (g/L)	0.1	0.5
X_15_	FeSO_4_. 7H_2_O (g/L)	0	0.01

**Table 2:** Twenty-trial Plackett–Burman experimental design for evaluation of fifteen independent variables with coded values along with the observed L-asparaginase activity.

Run	X_1_	X_2_	X_3_	X_4_	X_5_	X_6_	X_7_	X_8_	X_9_	X_10_	X_11_	X_12_	X_13_	X_14_	X_15_	Dummy _1_	Dummy _2_	Dummy _3_	Dummy _4_	L-asparaginase activity (U/mL)		Residuals
																				Experimental	Predicted	
1	1	−1	1	−1	−1	−1	−1	1	1	−1	1	1	−1	−1	1	1	1	1	−1	44.095	47.339	−3.244
2	−1	1	1	−1	1	1	−1	−1	1	1	1	1	−1	1	−1	1	−1	−1	−1	34.371	38.262	−3.891
3	1	−1	−1	−1	−1	1	1	−1	1	1	−1	−1	1	1	1	1	−1	1	−1	21.179	22.755	−1.576
4	1	1	1	1	−1	1	−1	1	−1	−1	−1	−1	1	1	−1	1	1	−1	−1	72.033	77.592	−5.559
5	1	−1	1	−1	1	−1	−1	−1	−1	1	1	−1	1	1	−1	−1	1	1	1	17.080	−3.891	3.891
6	−1	−1	−1	−1	1	1	−1	1	1	−1	−1	1	1	1	1	−1	1	−1	1	40.025	38.449	1.576
7	1	1	−1	1	1	−1	−1	1	1	1	1	−1	1	−1	1	−1	−1	−1	−1	25.108	24.606	0.502
8	−1	1	1	−1	−1	1	1	1	1	−1	1	−1	1	−1	−1	−1	−1	1	1	79.935	74.376	5.559
9	1	1	−1	1	−1	1	−1	−1	−1	−1	1	1	−1	1	1	−1	−1	1	1	42.587	37.028	5.559
10	−1	−1	−1	1	1	−1	1	1	−1	−1	1	1	1	1	−1	1	−1	1	−1	6.604	8.180	−1.576
11	−1	−1	1	1	−1	1	1	−1	−1	1	1	1	1	−1	1	−1	1	−1	−1	8.198	9.365	−1.166
12	1	1	−1	−1	1	1	1	1	−1	1	−1	1	−1	−1	−1	−1	1	1	−1	28.752	27.603	1.149
13	−1	−1	−1	−1	−1	−1	−1	−1	−1	−1	−1	−1	−1	−1	−1	−1	−1	−1	−1	34.331	33.829	0.502
14	−1	1	1	1	1	−1	1	−1	1	−1	−1	−1	−1	1	1	−1	1	1	−1	33.648	32.499	1.149
15	1	−1	−1	1	1	1	1	−1	1	−1	1	−1	−1	−1	−1	1	1	−1	1	22.944	25.761	−2.817
16	−1	1	−1	−1	−1	−1	1	1	−1	1	1	−1	−1	1	1	1	1	−1	1	7.515	10.332	−2.817
17	−1	−1	1	1	1	1	−1	1	−1	1	−1	−1	−1	−1	1	1	−1	1	1	20.325	19.159	1.166
18	1	−1	1	1	−1	−1	1	1	1	1	−1	1	−1	1	−1	−1	−1	−1	1	51.411	48.167	3.244
19	1	1	1	−1	1	−1	1	−1	−1	−1	−1	1	1	−1	1	1	−1	−1	1	14.518	15.667	−1.149
20	−1	1	−1	1	−1	−1	−1	−1	1	1	−1	1	1	−1	−1	1	1	1	1	56.479	56.981	−0.502

The “−1” sign correspond to the minimum value and the “+1” sign correspond to the maximum value of the input parameter range.

**Figure 3:**
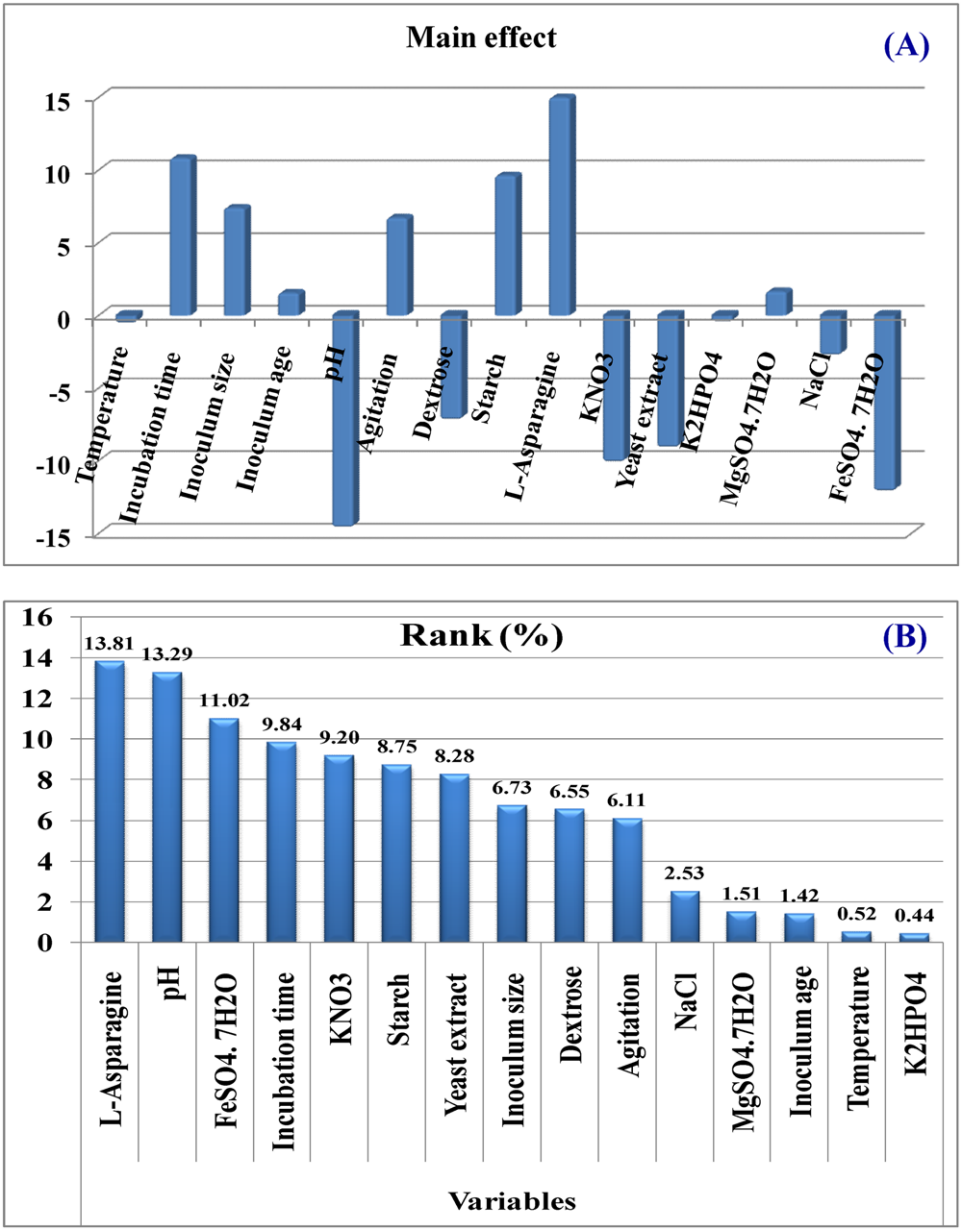
(**A**) The main effects of different variables on L-asparaginase production according to the Packett–Burman experimental results; (**B**) Pareto chart illustrates the order of significance of each variable.

The relationship between L-asparaginase production and the independent variables is determined by multiple-regression statistical analysis and the analysis of variance (ANOVA) of the experimental design was performed which is shown in Table 3. Table 3 and Fig. 3A show the estimated main effect of each variable on L-asparaginase production. With respect to the main effect (Fig. 3A), we can see that seven variables from the fifteen named L-asparagine, starch, incubation time, inoculum size, MgSO_4_.7H_2_O, agitation speed and inoculum age had positive effect on L-asparaginase production where the other eight variables had negative effect on L-asparaginase production (dextrose, K_2_HPO_4_, KNO_3_, yeast extract, NaCl, FeSO_4_.7H_2_O, pH and temperature). The order of significance of the variables that affect L-asparaginase production is illustrated by the Pareto chart (Fig. 3B).

**Table 3:** Statistical analysis of Plackett-Burman design showing coefficient values, *t*-test, *P*-values and confidence level (%) for each variable affecting L-asparaginase production by *Streptomyces brollosae* NEAE-115.

Variables	Coefficients	Main effect	*t* -Stat	*P*-value	Confidence level (%)
Intercept	26.50	53.00	29.32	0.0000	100.00
Temperature (°C)	−0.29	−0.58	−0.32	0.7655	23.45
Incubation time (days)	5.41	10.83	5.99	0.0039	99.61
Inoculum size (%, v/v)	3.70	7.41	4.10	0.0149	98.51
Inoculum age (h)	0.78	1.56	0.86	0.4364	56.36
pH	−7.32	−14.63	−8.10	0.0013	99.87
Agitation speed (rpm/min)	3.37	6.73	3.72	0.0204	97.96
Dextrose (g/L)	−3.61	−7.21	−3.99	0.0163	98.37
Starch (g/L)	4.82	9.64	5.33	0.0060	99.41
L-asparagine (g/L)	7.60	15.20	8.41	0.0011	99.89
KNO_3_ (g/L)	−5.06	−10.13	−5.60	0.0050	99.50
Yeast extract (g/L)	−4.56	−9.12	−5.05	0.0072	99.28
K_2_HPO_4_ (g/L)	−0.24	−0.49	−0.27	0.8004	19.96
MgSO_4_.7H_2_O (g/L)	0.83	1.67	0.92	0.4091	59.09
NaCl (g/L)	−1.39	−2.79	−1.54	0.1980	80.20
FeSO_4_. 7H_2_O (g/L)	−6.06	−12.13	−6.71	0.0026	99.74
**Analysis of variance (ANOVA)**					
	**Degree of freedom**	**Sum of squares**	**Mean sum of squares**	***F*** **- Fisher’s function**	**Significance F (** ***P*** **-value)**
Regression	15	5769.95	384.66	23.55	0.0038
Residual	4	65.33	16.33		
Total	19	5835.27			

Multiple R 0.9943, R Square 0.9888, Adjusted R Square 0.9468.

Significance of each coefficient was determined by *P*-values and student’s *t*-test, which are listed in Table 3. The smaller *P*-value and larger *t*-value means the more significant corresponding coefficient^13^. In the current experiment, variables evidencing *P*-values of less than 0.1 (confidence levels exceeding 90%) were considered to have significant effects on the activity of L-asparaginase. The ANOVA of the Plackett-Burman design demonstrated that the model was highly significant as was evident from the Fisher’s *F*-test value of 23.55 with a very low probability value (*P*-value = 0.0038) (Table 3) implies that the terms are significant.

By neglecting the terms that were insignificant (*P* > 0.1), the first order polynomial equation representing the production of L-asparaginase in terms of independent variables was derived:

1 $${{\bf{Y}}}_{(L-\mathrm{asparaginase}\mathrm{production})}={\rm{26.50}}+{\rm{5.41}}\times {\rm{incubation}}\,{\rm{time}}\,+\,{\rm{3.70}}\times {\rm{inoculum}}\,{\rm{size}}\,-{\rm{7.32}}\times {\rm{pH}}+{\rm{3.37}}\times {\rm{agitation}}\,{\rm{speed}}\,-{\rm{3.61}}\times {\rm{dextrose}}+{\rm{4.82}}\times {\rm{starch}}\,+{\rm{7.60}}\times L-\mathrm{asparagine}-{\rm{5.06}}\times {{\rm{KNO}}}_{{\rm{3}}}\,-\,{\rm{4.56}}\times {\rm{yeast}}\,{\rm{extract}}-\,{\rm{6.06}}\times {{\rm{FeSO}}}_{{\rm{4}}}{{\rm{.7H}}}_{{\rm{2}}}{\rm{O}}$$

### The adequacy of the model fit

**Figure 4:**
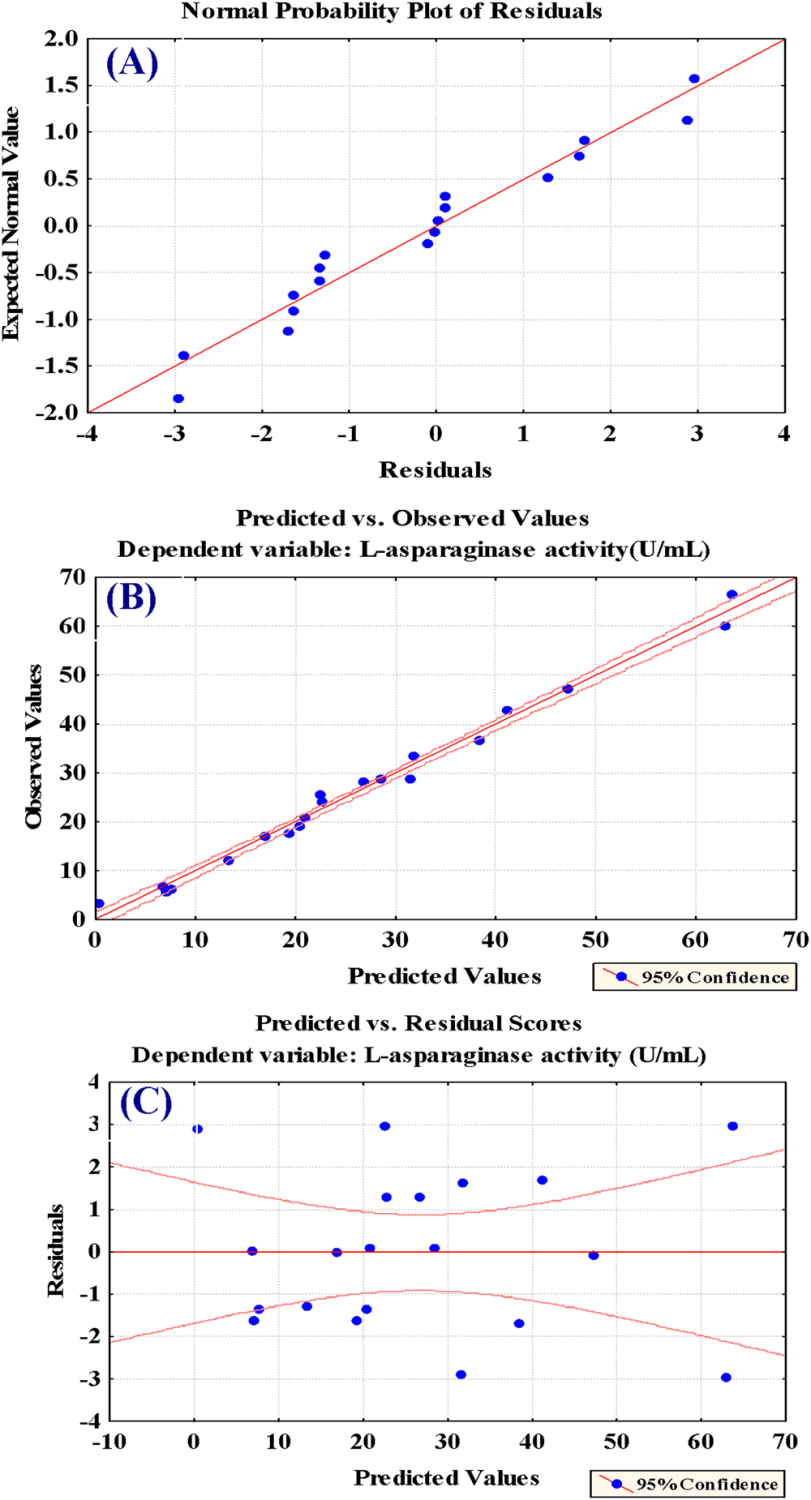
(**A**) The normal probability plot of residuals for L-asparaginase production as determined by the first-order polynomial equation, (**B**) correlation between predicted against observed values and (**C**) Plot of residuals against predicted values.

The normal probability plot of the residuals is an important graphical method detecting and explaining whether or not a data set is normal or departure from the normality^14^. Figure 4A shows residuals were plotted against the expected normal values of the model. The normal probability plot of the residuals shows the points near the diagonal line; so, the residuals are distributed normally. This indicates that the expected L-asparaginase production was well fitted with the experimental results. Figure 4B presents the plot of predicted values vs. observed values of the response. Points gathered around the diagonal line indicate a good correlation between the expected and experimental values. Figure 4C shows the plot of predicted versus the residuals, showing an equal distribution of residuals data above and below the x-axis, supporting the adequacy of the appropriate model.

In a confirmation experiment, to assess the accuracy of Plackett-Burman design, the conditions that are expected to be optimum for maximal production of L-asparaginase were pH 7, temperature 30 °C, inoculum size 4%, v/v, inoculum age 48 h, agitation speed 150 rpm/min, incubation time 7 days and medium of the following composition: g/L (dextrose 2, starch 15, L-asparagine 7, KNO_3_ 1, K_2_HPO_4_ 1, MgSO_4_.7H_2_O 0.5 and NaCl 0.1). Under these conditions, the maximum L-asparaginase activity was 75.151 U/mL which is higher than the L-asparaginase activity before applying Plackett Burman design (20.37 U/mL) by 3.69 times.

Erva *et al*.^15^ developed a system for optimizing culture conditions and nutritional components in order to enhance the production of extracellular antileukemic L-asparaginase by novel *Enterobacter aerogenes* KCTC2190/MTCC111. Traditional one variable at a time method was used to find the key culture conditions and nutritional components and then response surface methodology was implemented to determine their optimal concentrations. An enzyme activity of 18.35 U/mL was achieved at the optimum process variable values (incubation time was at 40 h, pH 6, temperature was maintained at 33 °C, substrate concentration was 1.34%, and inoculum size of 2%, V/V).

Baskar and Renganathan^16^ used the 2-level Plackett-Burman design in 12 experimental run to evaluate the importance of 8 process variables of medium components and operating conditions for the production of L-asparaginase by *Aspergillus terreus* MTCC 1782. The most important factors significantly influenced L-asparaginase production were L-asparagine, corn flour, urea and potassium chloride. Shakambari *et al*.^17^ used the Plackett-Burman design involving 12 trials and two levels of concentrations to evaluate the effect of different culture conditions on glutaminase free L-asparaginase production by *Pseudomonas plecoglossicida* RS1. They reported that, out of seven crucial factors examined, KH_2_PO_4_, sugar cane industry effluent as an alternate substrate to L-asparagine and pH were the most significant factors and the maximum L-asparaginase production was 3.63 U mL^−1^ in the optimized medium. As well, Kumar *et al*.^18^ used the Plackett-Burman design to evaluate the importance of 23 fermentation variables for L-asparaginase production by *Streptomyces radiopugnans* MS1. The most important variables were Tapioca effluent, corn steep liquor, L-asparagine and aeration. On the other hand, Venil *et al*.^19^ optimized the medium for production of L-asparaginase by *Serratia marcescens* SB08. They found that, out of 11 fermentation variables screened by Plackett-Burman design experiments, four variables, sucrose, peptone, KH_2_PO_4_ and incubation time, were the best for production of L-asparaginase. Moreover, Rajamanickam *et al*.^20^ used 12 runs Plackett-Burman design to screen the influence of eleven factors on L-asparaginase production by *Streptomyces parvulus* KUA106. Among the factors screened, four factors with the greatest significance effects were asparagine, tryptone, dextrose and NaCl.

### Statistical optimization of L-asparaginase production by *Streptomyces brollosae* NEAE-115 using face centered central composite design (FCCD)

The calculated *t*-values (Table 3) revealed that, incubation time (X_2_), starch (X_8_) and L-asparagine (X_9_) were the most significant factors had positive effects on L-asparaginase production by *Streptomyces brollosae* NEAE-115 and thus were selected for further optimization using FCCD. Other factors in this study were maintained at a constant level giving maximum production of L-asparagine in Plackett-Burman experiments. A total of 20 experiments with different combination of X_2_, X_8_ and X_9_ were performed and the experimental and predicted L-asparaginase production and residuals are presented in Table 4. The results showed significant variations in production of L-asparaginase. The maximum L-asparaginase activity (80.39 U/mL) was achieved in the center point runs (numbers 9, 10, 13, 14, 18 and 20) when using incubation time of 7 days, 20 g/L starch and 10 g/L L-asparagine. Whereas, the minimum L-asparaginase activity (7.52 U/mL) was observed in the run number 12 after 9 days of incubation time when10 g/L starch and 7 g/L L-asparagine were used.

**Table 4:** Face-centered central composite design with coded and actual levels of the variables representing L-asparaginase activity as influenced by X_2_ (incubation time), X_8_ (starch), X_9_ (L-asparagine) along with the predicted L-asparaginase activity and residuals.

Trials	Variables			L-asparagine activity (U/mL)		Residuals
	X_2_	X_8_	X_9_	Experimental	Predicted	
1	1	0	0	36.05	40.71	−4.66
2	0	1	0	70.16	73.08	−2.92
3	1	1	1	15.97	18.43	−2.47
4	0	0	−1	58.93	63.43	−4.49
5	1	1	−1	41.69	37.87	3.81
6	0	−1	0	59.62	66.48	−6.87
7	−1	−1	1	30.14	31.51	−1.37
8	−1	1	1	20.15	16.03	4.13
9	0	0	0	80.39	77.13	3.26
10	0	0	0	80.39	77.13	3.26
11	−1	0	0	34.27	39.40	−5.12
12	1	−1	−1	7.52	9.20	−1.68
13	0	0	0	80.39	77.13	3.26
14	0	0	0	80.39	77.13	3.26
15	−1	−1	−1	13.89	8.98	4.92
16	1	−1	1	21.24	16.24	5.00
17	0	0	1	59.68	64.97	−5.29
18	0	0	0	80.39	77.13	3.26
19	−1	1	−1	17.42	19.97	−2.55
20	0	0	0	80.39	77.13	3.26
**Level**	**(days)**	**(g/L)**	**(g/L)**			
−1	5	10	7			
0	7	20	10			
1	9	30	15			

### Multiple regression analysis and ANOVA

Multiple regression analysis was performed to analyze the data and the results of the analysis are presented in Table 5. Analysis of variance (ANOVA) is required for the quadratic regression model to test its significance and adequacy. The ANOVA (Table 5) demonstrates that the model is highly significant, as is evident from the Fisher’s *F*-test (48.89) and a very low probability value (4.33737E-07). The significance of each coefficient was determined by the values of *t* and *P* (Table 5).

**Table 5:** Statistical analysis of face-centered central composite design showing coefficient values, main effect, *t*-test and *P*-values.

Variables	Coefficients	Main effect	*t –* student’s test	*P*-Value	
Intercept	77.13	154.26	40.06	0.0000	
X_2_	0.66	1.31	0.37	0.7185	
X_8_	3.30	6.60	1.86	0.0921	
X_9_	0.77	1.55	0.44	0.6718	
X_2_X_8_	4.42	8.84	2.23	0.0497	
X_2_X_9_	−3.87	−7.75	−1.96	0.0790	
X_8_X_9_	−6.62	−13.24	−3.34	0.0075	
X_2_X_2_	−37.08	−74.15	−10.98	0.0000	
X_8_X_8_	−7.35	−14.69	−2.18	0.0547	
X_9_X_9_	−12.93	−25.85	−3.83	0.0033	
**Analysis of variance (ANOVA)**					
	**Degree of freedom**	**Sum of squares**	**Mean sum of squares**	***F*** **- Fisher’s function**	**Significance F (** ***P*** **-value)**
Regression	9	13804.60	1533.844	48.89	4.33737E-07
Residual	10	313.73	31.37		
Total	19	14118.32			

X_2_ the coded value of incubation time, X_8_ the coded value of starch and X_9_ the coded value of L-asparagine. Multiple R 0.9888, R Square 0.9777, Adjusted R Square 0.957.

In order to evaluate the relationship between L-asparaginase production and incubation time (X_2_), starch (X_8_) and L-asparagine (X_9_) and to calculate the maximum L-asparaginase production corresponding to the optimum levels of these variables, a second-order polynomial model equation was proposed to define the predicted response (Y) in terms of the independent variables (X_2_, X_8_ and X_9_):

2 $${{\rm{Y}}}_{(L-{\rm{a}}{\rm{s}}{\rm{p}}{\rm{a}}{\rm{r}}{\rm{a}}{\rm{g}}{\rm{i}}{\rm{n}}{\rm{a}}{\rm{s}}{\rm{e}}{\rm{p}}{\rm{r}}{\rm{o}}{\rm{d}}{\rm{u}}{\rm{c}}{\rm{t}}{\rm{i}}{\rm{o}}{\rm{n}})}=77.13+0.66\,{{\rm{X}}}_{2}+3.30\,{{\rm{X}}}_{8}\,+\,0.77\,{{\rm{X}}}_{9}+4.42\,{{\rm{X}}}_{2}{{\rm{X}}}_{8}-3.87\,{{\rm{X}}}_{2}{{\rm{X}}}_{9}\,-\,6.62\,{{\rm{X}}}_{8}{{\rm{X}}}_{9}-\,37.08\,{{\rm{X}}}_{2}^{2}-\,7.35\,{{\rm{X}}}_{8}^{2}-\,12.93\,{{\rm{X}}}_{9}^{2}$$

where X_2_, X_8_ and X_9_ are the coded values of incubation time, starch and L-asparagine; respectively.

### Three dimensional (3D) plots

**Figure 5:**
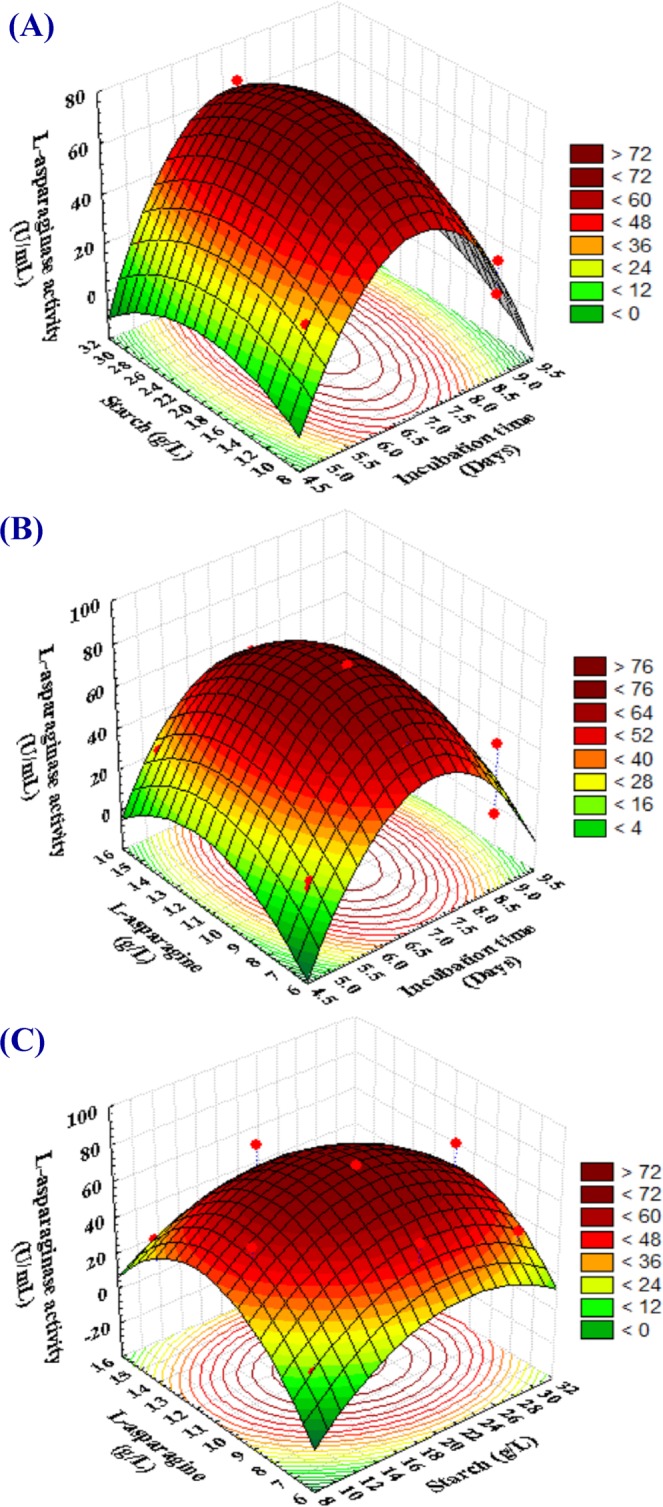
(**A**–**C**) Three-dimensional response surface plots showing the effect of incubation time (X_2_), starch (X_8_) and L-asparagine (X_9_) and their interactions effects on the production of L-asparaginase by *Streptomyces brollosae* NEAE-115.

The interaction effects and optimal levels of the variables were determined by plotting the three dimensional response surface curves (Fig. 5A–C) when one of the variables is fixed at optimum level and the other two are allowed to vary. Figure 5A represents the L-asparaginase activity as a function of incubation time (X_2_), starch (X_8_) by keeping L-asparagine (X_9_) at optimum value. Results showed that the lower and higher incubation time levels supported relatively low levels of L-asparaginase activity. The highest level of L-asparaginase activity was obtained with intermediate incubation time and starch concentration value. Incubation time has a significant effect on the production of L-asparaginase. The effect of incubation time on L-asparaginase production by *Streptomyces acrimycini* NGP was studied. Initial production of L-asparaginase on first day was 1.56 U/mL and increased up to 3.97 U/mL on 7^th^ day of incubation period^21^. Maximum growth and production of L-asparaginase (ranging from 186.37 to 257.06 U/mL) by *Serratia marcescens* SB08 showed at 51 h of incubation period and its growth declines in further extended incubation period. Extended incubation time might lead to the enzyme destruction due to interaction with other medium components^22^. L-asparaginase was produced by marine actinomycetes in incubation period of 96 hours^23^. In another report, L-asparaginase activity was increased with time to reach maximum value on the 5^th^ day of incubation^24^. Varalakshmi and Raju^25^ reported that fermentation after 96 hours showed a decrease in L-asparaginase production, which could be attributed to the disruption of the enzyme due to the presence of some kind of proteolytic activity, due to nutrients depletion and accumulation of toxic end products.

Carbon sources are used to promote growth and thus result in an increase in enzyme production, which is usually observed in the synthesis of primary metabolites, such as enzymes^26^. Glucose was considered as a catabolic repressor for L-asparaginase production by certain bacterial strains like *E. coli*. Synthesis of L- asparaginase in *E. coli* is almost completely suppressed if glucose is added at a concentration of 0.5% to the growth medium^27^. In contrast, another literature on L-asparaginase production by *Streptomyces* spp. reported that glucose followed by starch served as good carbon source for maximal production of L-asparaginase by *Streptomyces ginsengisoli*^10^. Also, the biosynthesis of L-asparaginase by *Streptomyces albidoflavus* was high when the strain was cultivated in basal medium with maltose as carbon source followed by starch, glucose, trehalose and glycerol^28^. *Bacillus* sp. WF67^29^, *Serratia marcescens*^30^ and *Bacillus* sp.^31^ preferred glucose as a carbon source to produce L- asparaginase maximally. Dextrose and starch positively affect the production of glutaminase free L-asparaginase by *Streptomyces olivaceus* NEAE-119^32^. L-asparagine or the combination of glucose and L-asparagine was found to be the best carbon sources for maximization of L-asparaginase production^33^.

Figure 5B represents the L-asparaginase activity as a function of incubation time (X_2_) and L-asparagine (X_9_) by keeping starch (X_8_) at optimum value. L-asparaginase activity was maximized at moderate to high levels of L-asparagine and moderate levels of incubation period, and the increase in incubation period resulted in a gradual decrease in L-asparaginase activity.

Venil and Lakshmanaperumalsamy^34^ reported that the addition of organic nitrogen sources in L-asparagine broth medium was found to induce maximum L- asparaginase production. Whereas, inorganic nitrogen supplements reduce L-asparaginase production significantly. L-asparagine works as a sole source of nitrogen and as inducer for the production of L-asparaginase. Therefore, its differing concentrations will have some effects on enzyme production. The optimum concentration of L-asparagine as a sole nitrogen source was determined to be 1% for L-asparaginase production by *Streptomyces* ABR2^35^. L-asparagine was reported to be the best nitrogen source for maximum production of L-asparaginase by *Streptomyces venezuelae* and *Streptomyces karnatakensis*^36^. Amena *et al*.^37^ reported that 0.5% L-asparagine proved to be the best nitrogen sources for L-asparaginase production by *Streptomyces gulbargensis*. Whereas, enhanced L-asparaginase production by *Streptomyces albidoflavus* was observed by using yeast extract as nitrogen source^28^.

Figure 5C showes that the maximum L-asparaginase production was attained beyond middle levels of starch (X_8_). Highest value of L-asparaginase production was obtained beyond high L-asparagine concentration (X_9_); lower and higher levels of L-asparagine resulted in a gradual decrease in L-asparaginase production.

### Verification of the model

To validate the accuracy of the model and to verify the result, an experiment was conducted under optimal conditions obtained from the face-centered central composite design and compared to the predicted data. The measured L-asparaginase activity obtained was 80.39 U/mL is close to the predicted one 77.13 U/mL revealing that a high degree of accuracy (95.94%).

Kumar *et al*.^18^ reported that the optimal levels of the four selected factors for maximum L-asparaginase production (19.5 U/mL) as obtained from Box-Behnken design were Tapioca effluent, 5% (v/v); L-asparagine, 0.003% (w/v); corn steep liquor, 2% (w/v); aeration, 0.7 vvm. Meena *et al*.^38^ used a Box-Behnken design based optimization to investigate the correlation between four variables (pH, starch, yeast extract and L-asparagine) and extracellular L-asparaginase production by marine actinobacteria, *Streptomyces griseus* NIOT-VKMA29. The optimal values of variables for the highest L-asparaginase production were determined to be: L-asparagine concentration 1.53%, starch concentration 1.48%, yeast extract concentration 2.04% and pH 8.04. Box-Behnken guided design of experiments resulted in a maximum L-asparaginase production of 56.50 (U/mL).

Shakambari *et al*.^17^ used central composite design (CCD) to optimize the levels and analyze the combined effect of KH_2_PO_4_, effluent and pH that were significant for L-asparaginase production by *Pseudomonas plecoglossicida* using the optimized medium inoculated with 2% (v/v) inoculum, incubated at 37 °C for 48 h in static conditions. The optimum levels of KH_2_PO_4_, effluent and pH for the highest L-asparaginase production (3.25 U/mL) were determined to be KH_2_PO_4_ (0.2 g/L), effluent (0.8 mL) and pH (6.5). RSM guided design of experiments resulted in a maximum L-asparaginase production of 3.25 U/mL with a 4.5 fold increase in enzyme activity compared to that obtained in the unoptimized medium (enzyme activity 0.73 U/mL).

Optimization of process variables using central composite design was performed to find out the optimum values of four fermentation variables. The maximum production of 257.55 U/mL L-asparaginase was achieved under flask conditions using the optimal levels for sucrose, peptone, KH_2_PO_4_ and incubation time were determined as 12.50 g/L, 4.5 g/L, 4.0 g/L and 51 h^19^. Morales-Gonzalez *et al*.^39^ used central composite design to optimize the levels and to study the effects of nutritional and growth factors that improve L-asparaginase production by *Kitasatospora atroaurantiaca*, *Streptomyces panaciradicis* and *Streptomyces griseoluteus*. The maximum experimental L-asparaginase production by *Kitasatospora atroaurantiaca* (28.18 U/mg protein) was obtained at the optimal levels for lactose concentration (1% w/v), L-asparagine + malt extract concentration (1% w/v), temperature (35 °C) and pH (7). The maximum experimental L-asparaginase production by *Streptomyces panaciradicis* (31.20 U/mg protein) was achieved at optimal levels of process variables (lactose concentration was 1% w/v, L-asparagine + malt extract concentration was 1% w/v, pH was 7, temperature was maintained at 35 °C). On the other hand, The CCD results for L-asparaginase production by *Streptomyces griseoluteus* identified the optimal values for lactose, L-asparagine + malt extract concentrations, temperature and pH were 0.5% w/v, 0.5% w/v, 27.5 °C, 6.5; respectively and the maximum predicted L-asparaginase activity was 30.72 U/mg protein.

Four factors with the greatest significant effects on L-asparaginase production by *Streptomyces parvulus* KUA106 were selected for optimization of process using central composite design to find out the optimum levels and to examine the combined effects of these factors. The optimal levels of the four selected factors for maximum L-asparaginase production (135 U/mL) as obtained using CCD were asparagine (0.05%), tryptone (0.5%), dextrose (5%) and NaCl (0.05%)^20^. Mangamuri *et al*.^40^ used one factor at a time and CCD to evaluate the effect of various physico-chemical factors on L-asparaginase production by *Streptomyces labedae* VSM-6. Maximum L-asparaginase production (8.92 U/mL) by *Streptomyces labedae* VSM-6 using conventional one factor at a time optimization was found after 6^th^ day of incubation in production medium supplemented with 1% L-asparagine, 1.5% starch, 1% yeast extract at initial pH 8 after 144 h of incubation. The optimized levels of the nutritional and cultural conditions for maximum L-asparaginase production (10.17 U/mL) by *Streptomyces labedae* VSM-6 were found to be pH (8), temperature 30 °C, incubation time (6 days), concentrations of yeast extract, starch, and L-asparagine 1, 1.5, 1%; respectively as determined by using central composite design.

### Effect of different stirring speeds on L-asparaginase production under uncontrolled pH condition

**Figure 6:**
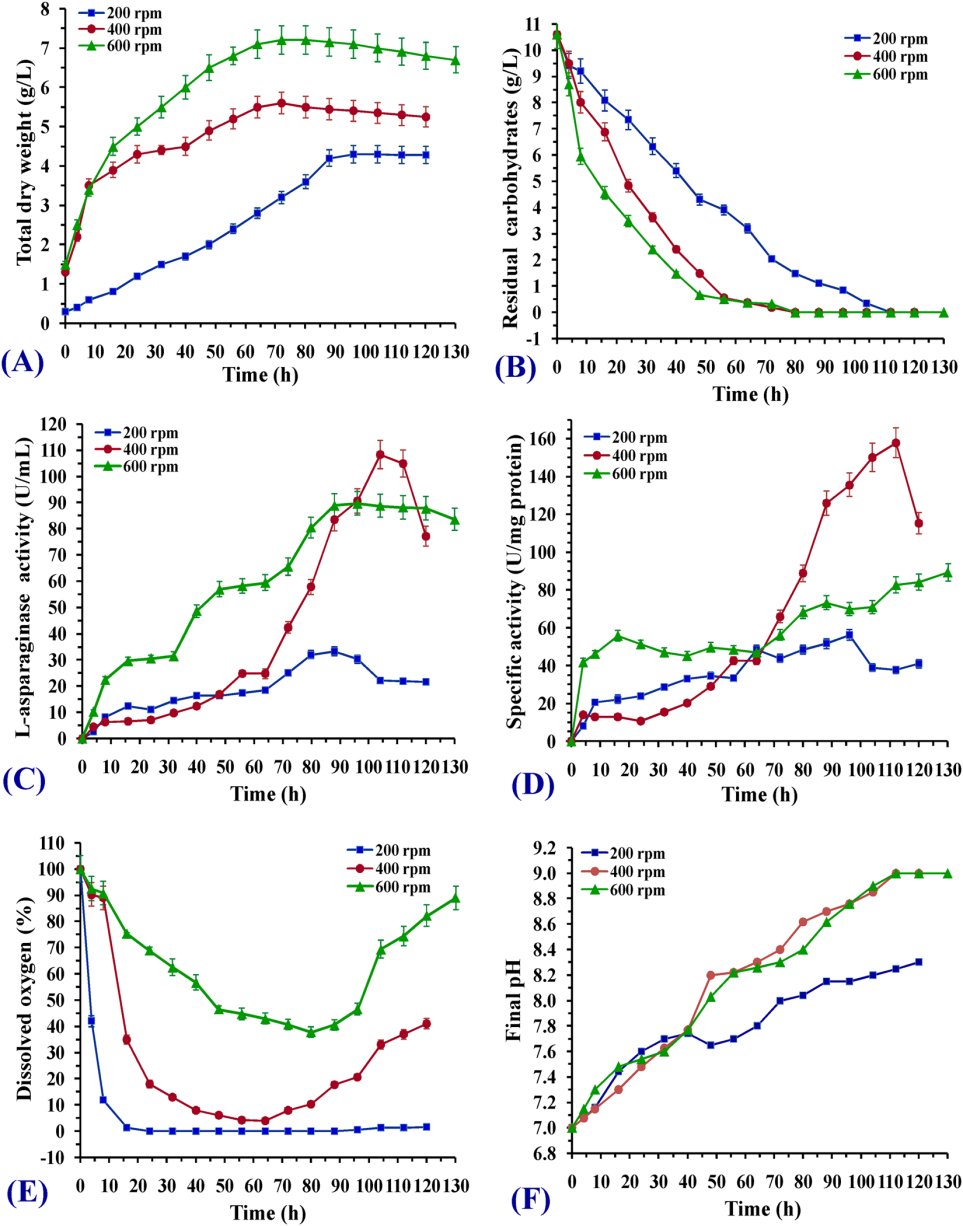
Time-course profile of (**A**) cell growth; (**B**) carbohydrates consumption; (**C**) L-asparaginase production; (**D**) specific activity; (**E**) dissolved oxygen and (**F**) final pH during batch cultivation of *Streptomyces brollosae* NEAE-115 in 7 L stirred tank bioreactor under different stirring speeds and uncontrolled pH.

To optimize the stirring speeds in the 7-L stirred tank bioreactor, three stirring speeds of 200, 400 and 600 rpm were adjusted before fermentation in the batch cultivation. The relations between cell growth, enzyme production and substrate consumption as a function of different stirring speeds were studied. As shown in Fig. 6A, the results indicate that the cell growth increased with the time in all cultures until reached a highest value then decreased slightly until the end of the fermentation period. In case of 200 rpm, cells grew exponentially for longer time up to 96 h at a rate [dx/dt] of 0.045 g/h^−1^ until reached [X_max_] 4.3 g/L. The specific growth rate [in h^−1^] which was calculated based on the exponential part of the growth phase was about 0.0225 h^−1^. Increasing the stirring speeds to 400 rpm resulted in an increase in cell growth rate recorded 0.067 g/h^−1^ until reached maximum value of 5.6  g/L at  76 h with specific growth rate of about 0.0343 h^−1^ (Table 6). On the other hand, in case of 600 rpm agitated culture, the maximal cell growth 7.2 g/L was detected after about 66 h of cultivation at a rate of 0.0864 g/h^−1^with specific growth rate of about 0.0383 h^−1^ (Fig. 6A). It was clearly observed that the specific growth rate increased with increasing the stirring speed. On the other hand, the time of the active growth of cells was shortened. During the exponential growth of cells in all cultures, carbohydrates were consumed gradually until depletion when maximum cell growth was recorded (Fig. 6B). In 200 rpm stirring speed, consumption rate of carbohydrates [Qs] was 0.1 g/h^−1^which is less than consumption rate of carbohydrates at 400 and 600 rpm (0.13 and 0.141 g/h^−1^; respectively).

In culture under 200 rpm stirring speed, enzyme production rate [Qp] of about 0.431 U/mL/h was observed during 80 h of cultivation after which a degradation in this rate with time by 0.366 U/mL/h until the end of the fermentation time was recorded. However, a longer production phase of the enzyme in 400 rpm reaching its highest value at 104 h with a production rate of 1.1 U/mL/h, after this time a decrease in enzyme production until 120 h as cells entered a stationary phase was detected. Despite the highest biomass in fermentation culture under higher stirring speed 600 rpm compared to 200 and 400 rpm, a moderate L-asparaginase amount attained its highest value of [P_max_] 89.7 U/mL at about 90 h (Fig. 6C) with a production rate of 0.98 U/mL/h which was calculated at the beginning of fermentation period. It was observed that maximal specific activity of the enzyme 157.9 (U/mg protein) at 112 h was recorded in culture of 400 rpm compared to other speed under study (Fig. 6D).

The data also revealed differences between the depletion in the dissolved oxygen which was significant at 200 rpm. During first 16 h of the course of fermentation, a fast and gradual decline of the DO% level was observed from 100% at the starting to 1.2% till reached 0% at 24 h whereas a reduction to 4% after 60 h at 400 rpm was noticed. In contrast, the depletion was insignificant at higher agitation rate (600 rpm). In all fermentation cultures under study, during the growth phase, the DO% decreased and reached a minimal value and then increased gradually thereafter as a function of the cell-growth termination when the carbohydrates were completely consumed (Fig. 6B,E).

It was also noticed that the pH of all cultures increased gradually after inoculation parallel to L-asparaginase production, reached its highest value of 8.3 at 120 h in case of stirring speed 200 rpm and 9 in case of 400 and 600 rpm at 112 h and 104 h; respectively (Fig. 6F). Since L-asparaginase converts L-asparagine to L-aspartic acid and ammonia which shifts the pH towards alkaline and the enzyme as noticed through the results obtained from stirred tank bioreactor at different stirring effect was growth linked and increasing the stirring speeds from 200 to 600 resulted in an increase in cell growth rate. On the other hand, the time of the active growth of cells was shortened. As it was reported before that maximum biomass production could also be correlated with high levels of L-asparaginase production. L-asparaginase formation showed a firm link to the active cell growth (Savitri *et al*.)^5^, so, at 200 rpm the time of active cell growth was longer than in higher speed, production of the enzyme was slower and conversion of all the L-asparagine in the medium was delayed and alkaline pH due to ammonia was weak contrary to the other higher stirring speeds.

**Figure 7:**
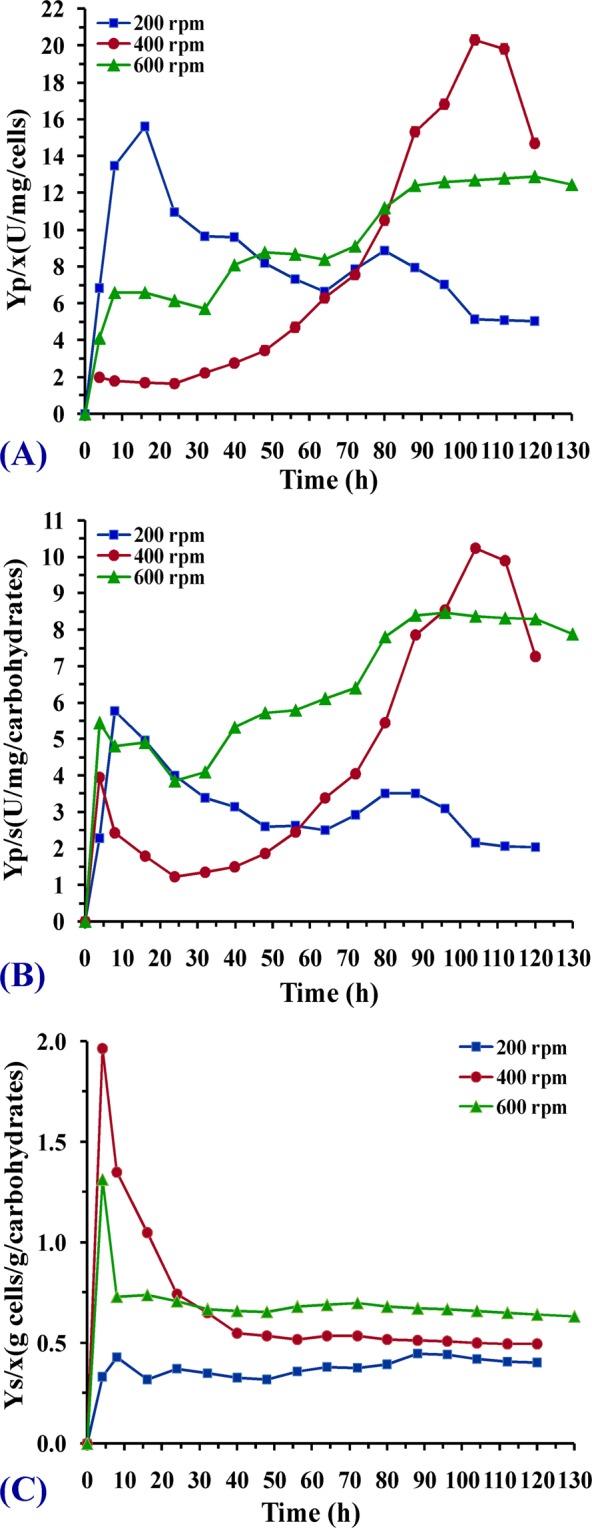
Time profile of yield coefficients: (**A**) (Yp/x) unit of L-asparaginase per mg biomass; (**B**) (Yp/s) unit of L-asparaginase per mg carbohydrates; (**C**) (Yx/s) g cells per g carbohydrates in batch cultivation under different stirring speeds in 7-L stirred tank bioreactor.

To clarify the cell performance toward L-asparaginase production in all cultures, the yield coefficients including Y_p/x_ (L-asparaginase produced/biomass of cells produced), Y_p/s_ (L-asparaginase produced/mass of carbohydrates consumed) and Y_x/s_ (biomass of cells produced/mass of carbohydrates consumed) were also calculated (Fig. 7). It was observed that the maximal value of the production yield coefficient Y_p/x_ 20.3 U/mg cells was in case of stirring speed 400 whereas lower values of Y_p/x_ (12.63 U/mg and 7.93 U/mg) were obtained at 600 and 200 rpm respectively. On the other hand, the highest Y_p/s_ of 10.23 U/mg carbohydrates value was obtained in case of 400 rpm followed by 3.5 and 8.46 U/mg carbohydrates in case of 200 and 600 rpm. These results indicated that in batch cultivation at 400 rpm the produced cells were more active towards enzyme production as the production yield coefficient was the highest compared to cultures under stirring at 200 and 600 rpm. Calculated coefficient biomass yield over substrate Y_x/s_ was the highest at the stirring speed of 600 rpm, i.e. 0.7 g biomass produced per g carbohydrates which was depleted after 66 h of cultivation whereas 0.54 and 0.44 g biomass produced per g carbohydrates were recorded in 400 and 200 rpm; respectively accompanied by carbohydrates depletion after 76 and 112 h. From these results, the enzyme activity at 400 rpm was the highest and obtained later by almost 8 h compared to the other speeds. Besides, the production yield coefficient was the highest hence, a stirring speed of 400 rpm is chosen as the optimal one for maximum biosynthesis of L-asparaginase.

One of the important factors that can affect microbial productions in scale-up is the agitation which influence directly on oxygen transfer rate in different scales. In this context, the effect of stirring speed and pH control on cell growth, L-asparaginase production and other parameter such as DO% was determined during the course of the submerged fermentation in batch cultivation of the producer microorganism *Streptomyces brollosae* NEAE-115 in 7-L stirred tank bioreactor. Due to the scarce specific literature available, comparison of our results with those of other scientists is difficult. The low cell biomass and enzyme activity at lower stirring speed (200 rpm) could be attributed to the shortage of oxygen being experienced by the organism due to the insufficient mixing. This agree with the results obtained by Priya *et al*.^41^, who reported that a positive correlation between L-asparaginase production by *Streptomyces* sp. (TA22) and cell mass over a time period of 24–120 hours. The microorganism at 600 rpm grew faster and got sheared more easily led to the access of maximum cell biomass earlier; at the same time it enhances shearing of cells which badly affect enzyme synthesis. In addition, the shear forces could partially inactivate the enzyme, already released in the cultural broth. In this study, low enzyme activity at low stirring speed at 200 rpm and stirring speed at 600 rpm could be related to catalytic dysfunctions due to conformational changes carried out by mechanical forces^42^. It was observed that as the stirring speed increased, cell biomass increased too because more oxygen was incorporated into the medium and lead to progressive accompanied by an acceleration of carbohydrates consumption. Once carbohydrates were depleted in the culture, a significant decrease in cell dry weight was observed. This reduction was a function of cell lysis under the combined effect of carbohydrates limitation and shear stress effect in the culture^43^. L-asparaginase production was higher in 400 rpm batch culture than in 200 stirring speed, this may be attributed to the dispersion of macromolecules in the medium and increase amount of dissolved oxygen. It might, therefore, contribute to the greater growth and extracellular protein value. A correlation between agitation speed and enzyme production has been reported by researches who worked on *Streptomyces gulbargensis*^44^. Results also showed that during all batch cultivations, cell growth is associated with large increase in oxygen uptake. In this study, during the active growth phase, the DO% in culture at 200 rpm dropped significantly after 24 h. In previous studies, it has been reported that DO% was gradually increased until the end of cultivation time when the cells entered the stationary phase^45^. On the other hand, during the fermentation time, it was noticed that pH of the medium increased gradually from the beginning of the fermentation time parallel to L-asparaginase production in all batch cultures reached 9 at higher agitation speed and 8.5 at 200 rpm.

### Batch cultivation for L-asparaginase production under controlled pH

**Figure 8:**
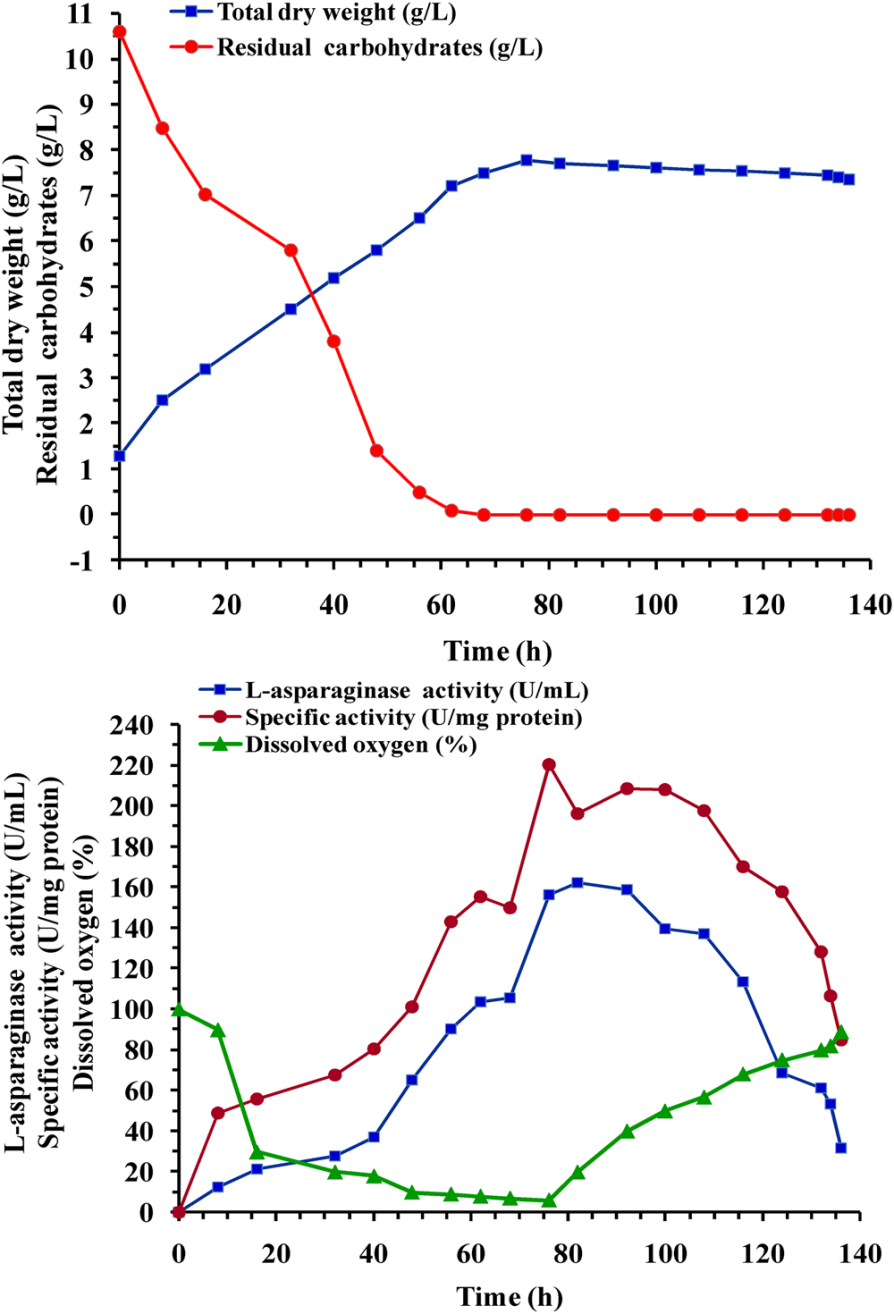
Time-course profile of cell growth; L-asparaginase production; carbohydrates consumption; specific activity and dissolved oxygen during batch cultivation of *Streptomyces brollosae* NEAE-115 in 7-L stirred tank bioreactor at stirring speed of 400 rpm and controlled pH 7.

**Figure 9:**
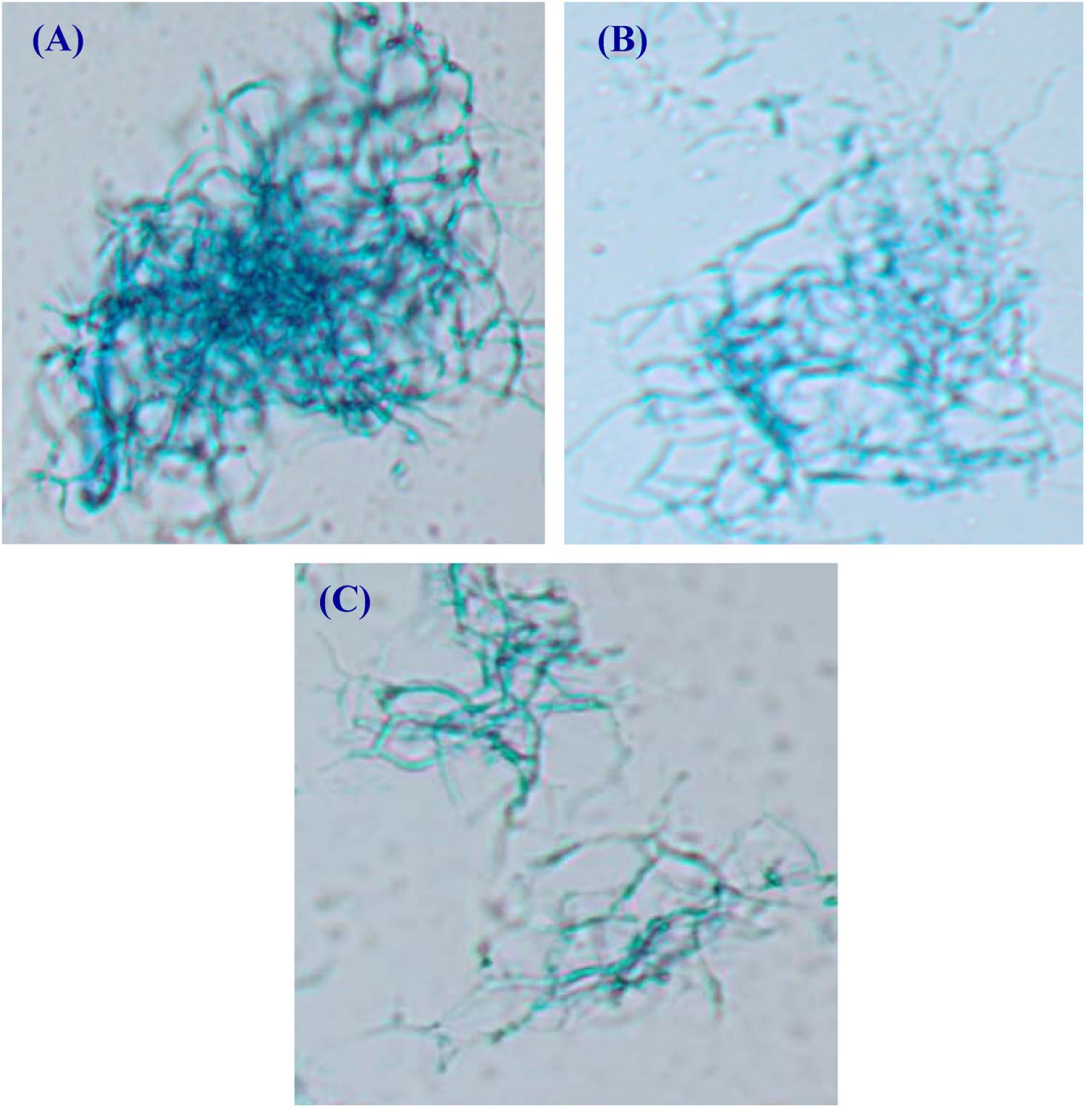
Microscopic observation of *Streptomyces brollosae* NEAE-115 cells at (**A**) early fermentation time showing mycelia clump, (**B**) dispersed mycelia at middle fermentation time and (**C**) freely dispersed mycelia and spores at late fermentation time of batch cultivations in 7-L stirred tank bioreactor.

The kinetic data of cell growth, carbohydrates consumption, enzyme activity and DO% during batch cultivation with a controlled pH 7 at 400 rpm was presented in Table 6 and Figs 8, 9. During the growth phase, cells grew exponentially with rate higher than in batch fermentation at 200 and 400 rpm under uncontrolled pH and almost the same as 600 rpm while the specific growth rate was 0.0316 h^−1^ and the cell biomass reached its highest value of 7.78 g/L at 76 h (Table 6). On the other hand, carbohydrates were consumed at a rate of 0.16 g/L/h. L-asparaginase production started from the first hours of fermentation time with a production rate of 1.99 U/mL/h and recorded its maximal activity of 162.11  U/mL at 82 h then a decrease in production was detected by a degradation rate of 3.3 U/mL/h until the end of fermentation time. Whereas, dissolved oxygen level falls rapidly when cell grows enter in the exponential phase. To clarify the difference in growth and production kinetics between cultures at 400 rpm in case of uncontrolled and controlled pH, kinetic parameters related to cell growth and enzyme activity were compared (Table 6).

**Table 6:** Kinetic parameters for cell growth, L-asparaginase production, carbohydrates consumption during *Streptomyces brollosae* NEAE-115 growth in 7-L stirred tank bioreactor under stirring speed of 400 rpm.

Kinetic Parameters	Stirring speed, 400	
*Streptomyces brollosae* NEAE-115 growth	Uncontrolled pH	Controlled pH
X _max-biomass_ (g/L)	5.6	7.78
X _max-time_ (h)	76	76
dx/dt (g/h^−1^)	0.067	0.0855
μ (h^−1^)	0.0343	0.0316
L-asparaginase production		
P_max-vol_ (U/mL)	108.46	162.11
P_max-time_ (h)	104	82
Q_p_ (U/mL/h)	1.1	1.99
Y_p/x_ (U/mg cells)	20.3	21
Specific activity (U/mg protein)	157.9	220.6
Carbohydrates utilization		
Q_s_(g/L/h)	−0.1324	−0.16
Y_x/s_ (g cells/g)	0.54	0.73
Y_p/s_(U/g cells)	10.23	15.3

“X _max-biomass_, maximal cell dry weight; dx/dt, cell growth rate; P_max-vol_ maximal L-asparaginase production; P_max-time_, time of maximal L-asparaginase production; Q_p_, L-asparaginase production rate; Y_p/x_, coefficient production yield over biomass; Q_s_,carbohydrates consumption rate; Y_x/s_ coefficient biomass yield over substrate;Y_p/s_, coefficient production yield over substrate”.

The presented data of cells cultivation under uncontrolled and controlled pH condition revealed that in both cultures, the dissolved oxygen levels were started with 100% and reached its minimum percentage at 76 h parallel to maximum cell growth. It is apparent that, cell grew exponentially in both cultivations in which there was no lag phase with different rates. However, controlled pH condition was more favorable for L-asparaginase and cell mass production than uncontrolled pH bioreactor. These differences in growth rate and cells yield are linked to the rate of carbohydrates consumption, which also reflects the cell physiology status activity.

When controlling the pH at 7 during cultivation, the cell mass increased about 1.4-fold when compared with that from the uncontrolled bioreactor cultures. The specific growth rate [μ] for the controlled bioreactor culture was also higher. A maximum L-asparaginase production (162.11 U/mL) by *Streptomyces brollosae* NEAE-115 was reached in pH controlled culture at 400 rpm which is not reported for any other actinomycete strain so far.

## Materials and Methods

### Microorganism and cultivation conditions

The new isolate previously isolated and identified as *Streptomyces brollosae* NEAE-115^46^ is a potent L-asparaginase-producing strain; cultured and maintained on plates containing starch nitrate agar medium; the inoculated plates were incubated at 30 °C for 7 days^12^.

### Detection of L-asparaginase production by plate assay

Detection of the potential production of L-asparaginase by *Streptomyces brollosae* NEAE-115 was performed according to the method of El-Naggar *et al*.^12^ with the use of asparagine dextrose salts agar (ADS agar) medium^47^.

### Inoculum preparation for submerged fermentation in shake flasks

Inoculums for submerged fermentation in shake flasks were prepared according to the procedure used by El-Naggar *et al*.^12^.

### L-asparaginase production by *Streptomyces brollosae* NEAE-115 using submerged fermentation in shake flasks

*Streptomyces brollosae* NEAE-115 was cultured in 250 mL Erlenmeyer conical flasks containing fifty mL of asparagine dextrose salts broth medium (at a specified pH). The inoculated flasks were incubated at 30–37 °C with shaking at 100–150 rpm on a rotatory shaker incubator. After the specified incubation period for each set of experimental trials, the mycelial growth of *Streptomyces brollosae* NEAE-115 was collected using cooling Centrifuge for 20 min at 5000 × *g* and 4 °C.

### Impact of some carbon sources on L-asparaginase production

A preliminary study was conducted to determine the impact of some carbon sources on L-asparaginase production by *Streptomyces brollosae* NEAE-115. Asparagine dextrose salt broth^47^ supplemented with different carbon sources such as dextrose, maltose, starch, sucrose and glycerol was used.

### Assay of L-asparaginase activity

The activity of L-asparaginase was determined by the direct nesslerization method by measuring the quantity of released ammonia^48^. The reaction mixture containing 0.5 mL of L-asparaginase and 1.5 mL of 0.04 M L-asparagine (prepared in 0.05 M Tris-HCl buffer, pH 8.6). The reaction mixture tubes were incubated in a water bath shaker at 37 °C for 30 minutes. 0.5 mL of 1.5 M Trichloroacetic acid (TCA) was used to stop the reaction. The blank was prepared by adding the enzyme after addition of TCA, one blank has been conducted for each sample tested. By centrifugation at 10,000 × *g* for 10 min, the precipitated proteins were separated. The released ammonia in the supernatant was colorimetrically determined by direct nesslerization by adding 1 mL of Nessler’s reagent to tubes containing 0.5 mL of clear supernatant diluted with 7 mL of distilled water and incubated at room temperature for 20 min. The yellow color formation indicates the presence of ammonia and was measured with UV-visible spectrophotometer (Optizen Pop–UV/Vis spectrophotometer) at 480 nm. The quantity of ammonia released was calculated using the standard curve of ammonium chloride. One unit (U) of L-asparaginase is defined as the amount of L-asparaginase that has catalyzed the formation of 1 µmole of ammonia from L-asparagine per minute under the standard assay conditions. The activity of the enzyme has been expressed as units per gram of dry fermented substrate (U/gds).

### Selection of significant variables influencing L-asparaginase production by Plackett–Burman design

The Plackett–Burman statistical experimental design^49^ is a two factorial design, which identifies different process variables that had a significant effect, either positively or negatively on L-asparaginase production during submerged fermentation in shake flasks with respect to their main effects^50^. Different process variables influencing L-asparaginase production by *Streptomyces* NEAE-115 were identified using Plackett–Burman experimental design. Total of fifteen variables (assigned) (dextrose, starch, L-asparagine, KNO_3_, yeast extract, K_2_HPO_4_, MgSO_4_.7H_2_O, NaCl and FeSO_4_. 7H_2_O, pH, incubation time, temperature, inoculum age, inoculum size and agitation speed) (Table 1) and four dummy (unassigned) variables were selected for this study in 20 trials (Table 2). Each variable is represented at high (+) and low (−) levels. The design of the Plackett-Burman depends on the first order model:

3 $$Y={\beta }_{0}+\sum {\beta }_{i}{X}_{i}$$

where, Y is the predicted L-asparaginase production, X_i_ is independent variable level, β_i_ is the linear coefficient and β_0_ is the model intercept. All runs were conducted in duplicate and the average of L-asparaginase activity was used as responses.

### Optimization of L-asparaginase production using face centered central composite design (FCCD)

The FCCD was used to determine the optimal levels of the three most significant positive independent variables affecting the production of the enzyme (incubation time, starch and L-asparagine) and to study the individual and mutual interactions among the tested variables on L-asparaginase production. The FCCD is a statistical experimental design in which each variable is varies on three different levels, low (−1), medium (0), high (+1) and the center point was repeated six times, resulting in a total of 20 runs. Given all linear, quadratic and interaction coefficients, the regression model can be illustrated using the following second-degree polynomial equation:

4 $$Y={\beta }_{0}+\sum _{i}{\beta }_{i}{X}_{i}+\sum _{ii}{\beta }_{ii}{X}_{i}+\sum _{ij}{\beta }_{ij}{X}_{j}$$

In which *Y* is the predicted response, β_0_ is the regression coefficients, β_i_ is the linear coefficient, β_ii_ is the quadratic coefficients, β_ij_ is the interaction coefficients), and X_i_ is the coded level of independent variable.

### Statistical analysis

The obtained experimental results were subjected to multiple regression analysis using Microsoft Excel, 2007 and the STATISTICA software “Version 8.0, StatSoft Inc., Tulsa, USA” was used to plot the three-dimensional surface plots.

### Inoculum preparation for 7-L stirred tank bioreactor

A first-stage seed culture was grown by inoculating a 250 mL shake flask (50 mL working volume) containing production medium composed of (g/L): (dextrose 1, soluble starch 5, KNO_3_ 1, yeast extract 1, K_2_HPO_4_ 2, MgSO_4_. 7H_2_O 0.5, NaCl 0.5 and FeSO_4_. 7H_2_O 0.01 with a single vial of frozen stock culture. The flask was incubated at 30 °C in a temperature controlled shaking incubator operating at 150 rpm for 48 h. A second stage of seed growth is required to prepares the microorganism for enzyme production. From the first-stage culture 5% (v/v) inoculum was transferred to a 1-L shake flask (250 mL working volume) containing the previously mentioned production medium, then incubated at 30 °C and 150 rpm for 48 h.

### Submerged fermentation using 7-L stirred tank bioreactor

Batch fermentations were inoculated by transferring around 10% (v/v) of the second stage flask culture directly to the 7-L stirred tank bioreactor (bioflow 310; New Brunswick Scientific, Edison, NJ, U.S.A.) containing the previously mentioned production medium (pH 7). The useful volume used in the fermentations was 5-L. The bioreactor is equipped with digitally controlled pH electrode, temperature probe, polarographic DO electrode “Ingold, Mittler-Toledo, Switzerland) and two six-blade Rushton turbine impellers (5.2 cm diameter)”, fixed on the agitator shaft above 3.2 cm air sparger. The pH electrode was calibrated by using standard buffers (Fluka) at pH 7 and 9 prior to the sterilization of bioreactor (121 °C for 20 min). However, the calibration of DO electrode was conducted after sterilization by the air until 100% saturation was achieved. The foam was manually controlled by adding a few drops of silicon -based antifoam (Sigma) at foam time.

The temperature in the 7-L vessel was controlled at 30 °C. The airflow rate was set at (0.5 vvm) using filtered sterile air and stirring speed was allowed by varying the stirring speed rate in the bioreactor (200, 400 and 600 rpm) for the comparison between different stirring speeds on the enzyme production without pH control. The suitability of the stirring speed was determined on the basis of the results obtained. The enzyme production was then studied by controlling the pH of the culture at 7.0 with sodium hydroxide 2 M or 2 M hydrochloric acid during the whole fermentation process.

### Sample preparation and determination of cells dry weight

10 mL of broth samples were collected at different time’s intervals during the fermentation process in pre-weighed, 15 mL sterile falcon tubes and centrifuged at 1865 RCF for 15 min. A small fraction of the supernatant was frozen at −20 °C for carbohydrates, protein and L-asparaginase activity determinations. Whereas, the pellets containing cell debris were washed twice with distilled water, centrifuged and then the washed pellets were dried to constant weight at 70 °C for determination of cells dry weight.

### Estimation of total protein

Total protein in culture filtrate was determined using bovine serum albumin as standard according to the method of Lowry *et al*.^51^.

### Estimation of total carbohydrates

The total carbohydrates were determined spectrophotometrically according to the method of Dubois *et al*.^52^.

## Conclusion

As a conclusion, maximum L-asparaginase production can be achieved at 400 rpm under controlled pH at 7. The fundamental results obtained in this research are beneficial for further development of *Streptomyces brollosae* NEAE-115 cultivation strategy for the overproduction on a pilot scale.
